# Supplementary figures and images for: ahctf1 and kras mutations combine to amplify oncogenic stress and restrict liver overgrowth in a zebrafish model of hepatocellular carcinoma
Source: eLife. 2023 Jan 17;12:e73407. doi: 10.7554/eLife.73407 (PMC9897728; doi:10.7554/eLife.73407)

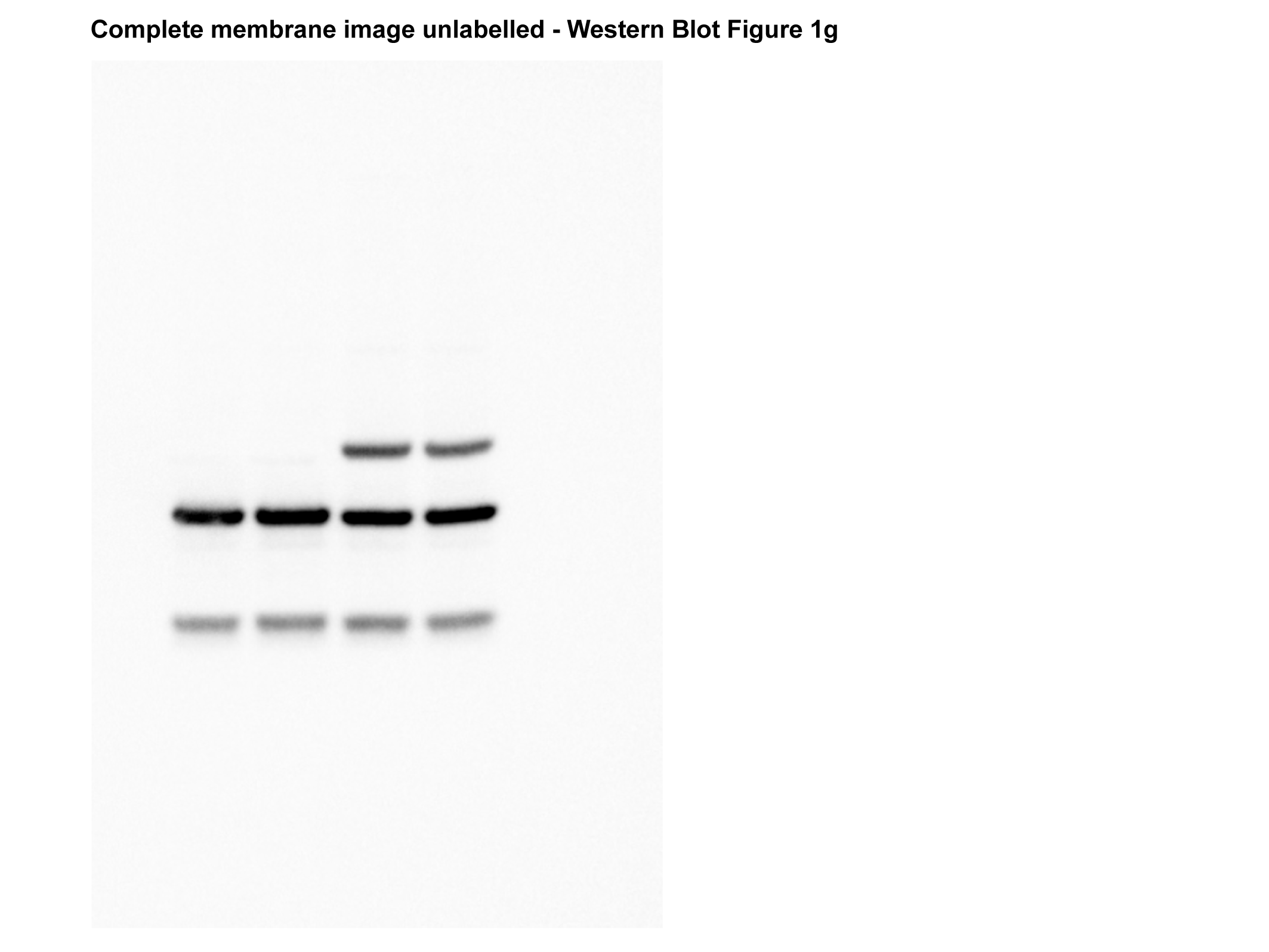

Supplement: Figure 1—source data 2. [file elife-73407-fig1-data2.zip › Figure 1-Source Data 2 uncropped unlabelled.tif]

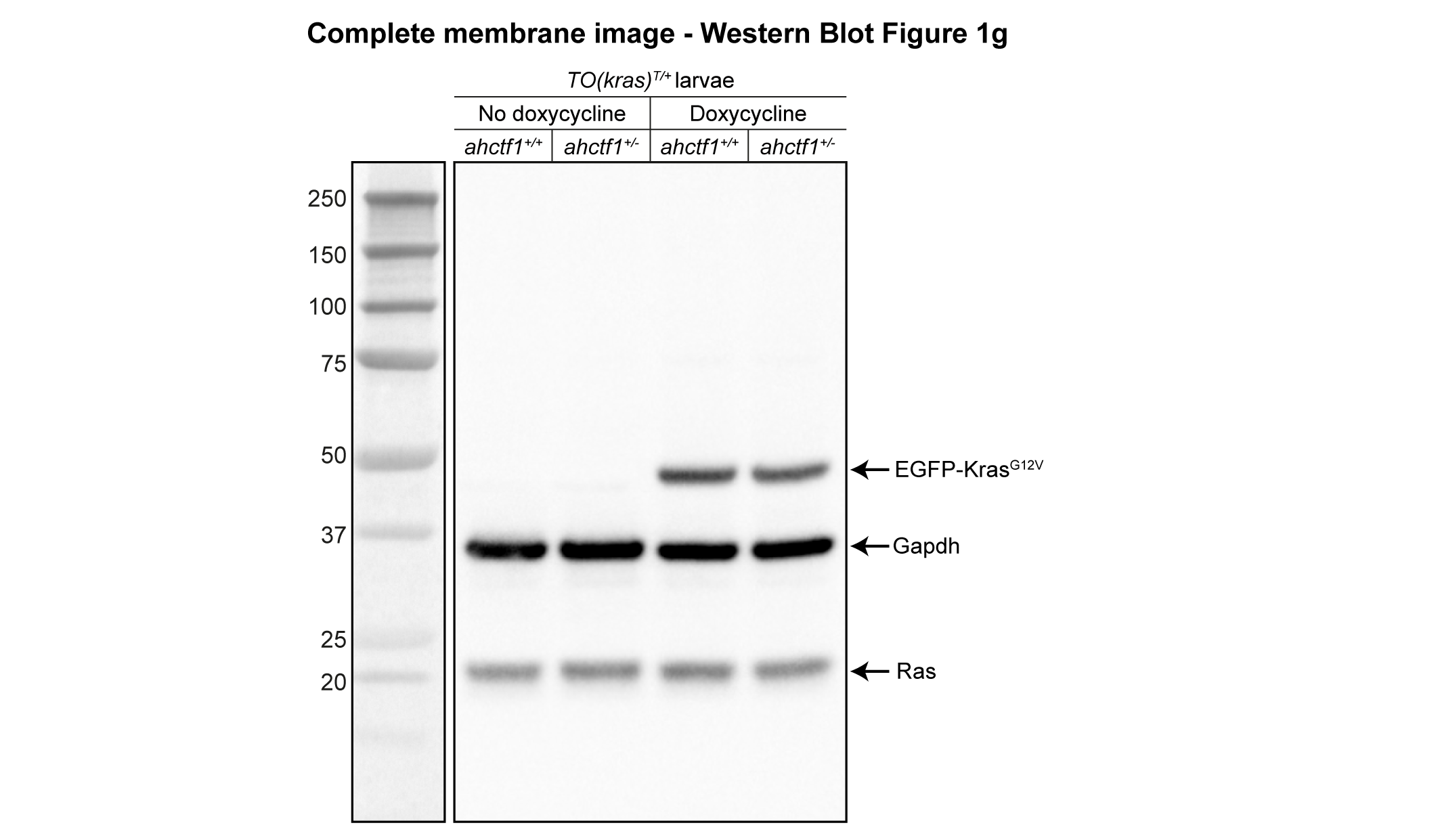

Supplement: Figure 1—source data 3. [file elife-73407-fig1-data3.zip › Figure 1-Source Data 3_uncropped panel 1g.tif]

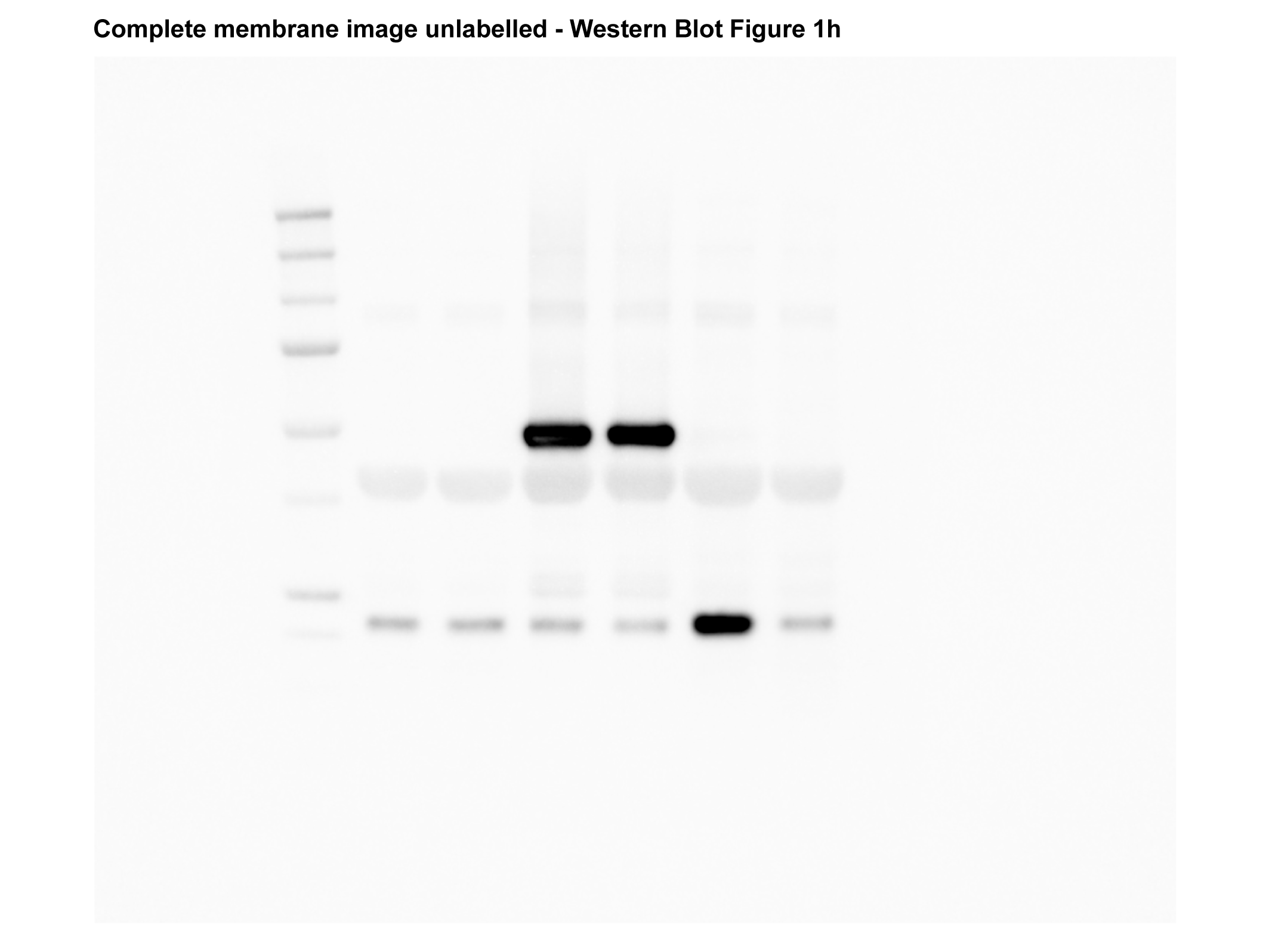

Supplement: Figure 1—source data 4. [file elife-73407-fig1-data4.zip › Figure 1-Source data 4 uncropped unlabelled.tif]

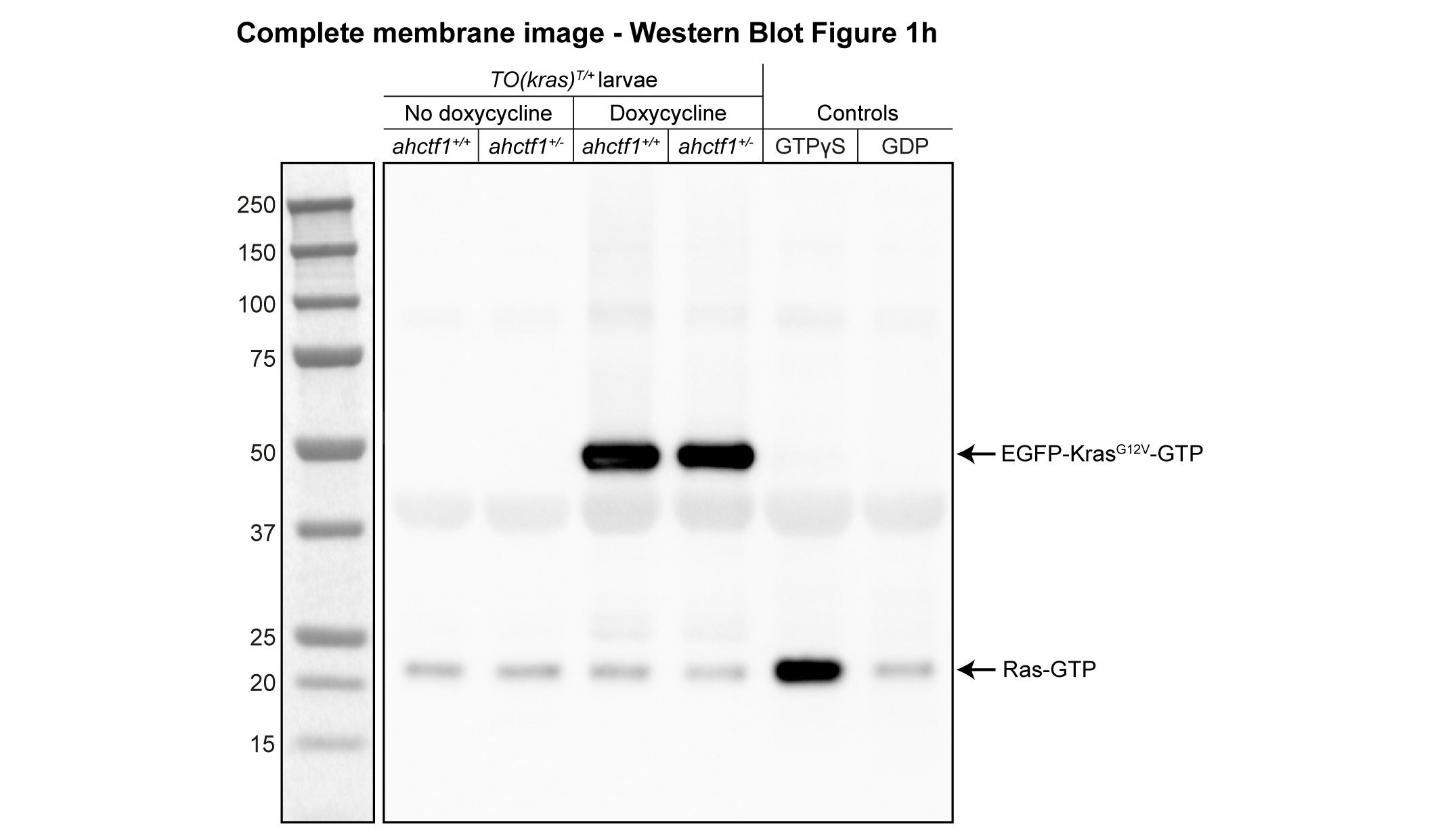

Supplement: Figure 1—source data 5. [file elife-73407-fig1-data5.zip › Figure 1-Source Data 5_uncropped panel 1h.tif]

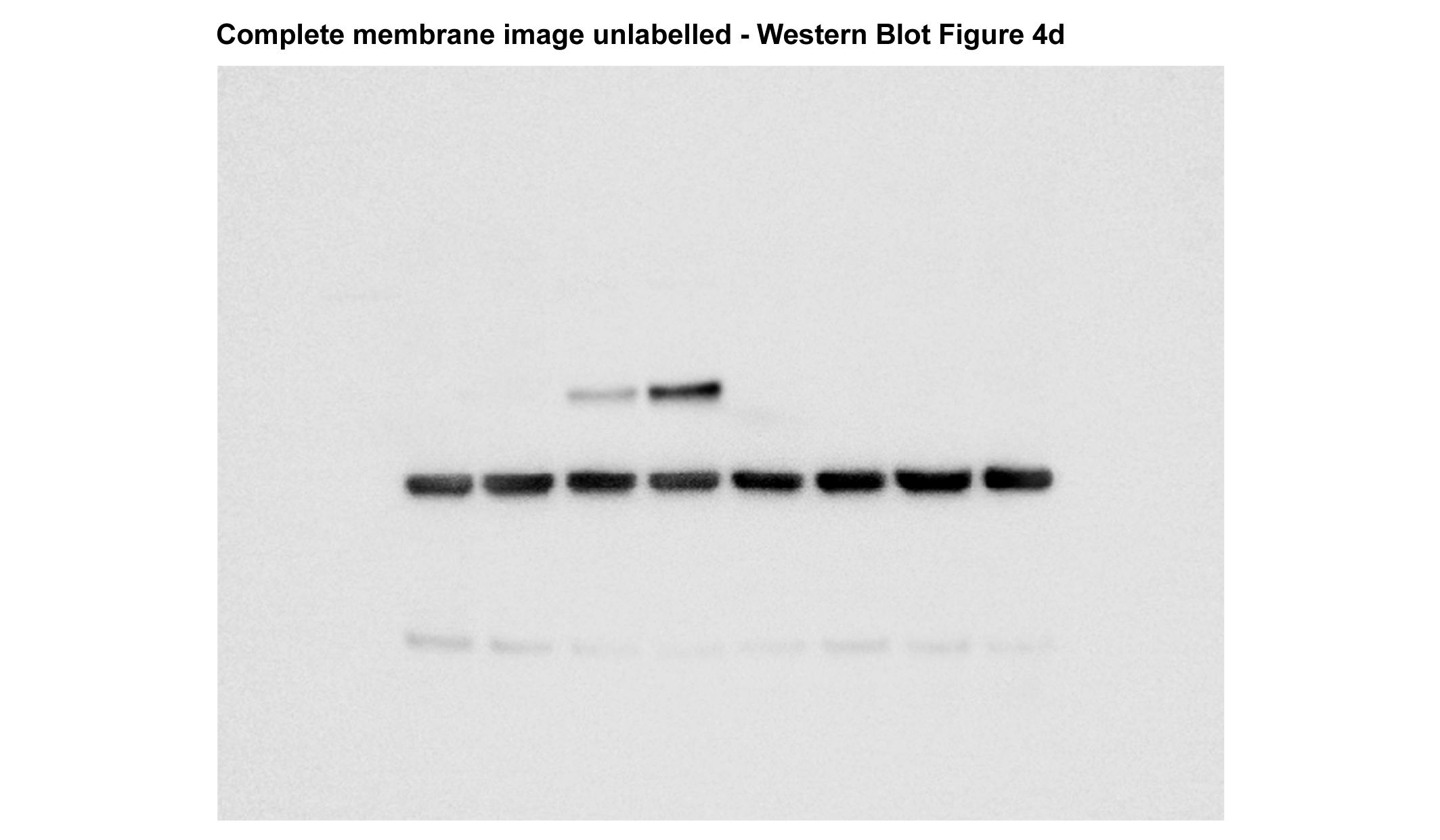

Supplement: Figure 4—source data 2. [file elife-73407-fig4-data2.zip › Figure 4-Source Data 2 uncropped and unlabelled.tif]

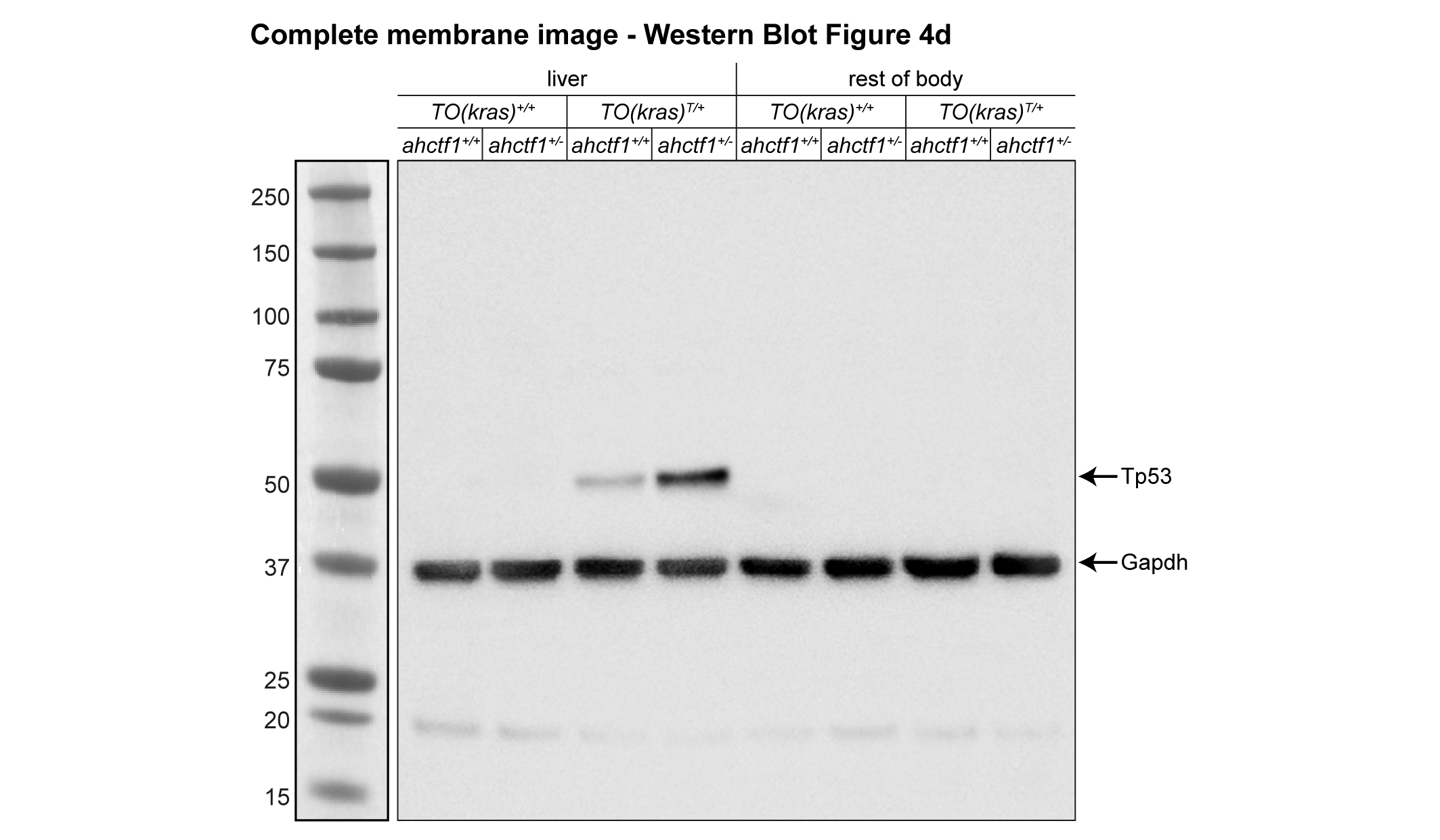

Supplement: Figure 4—source data 3. [file elife-73407-fig4-data3.zip › Figure 4-Source Data 3_uncropped panel 4d.tif]
