# Supplementary material for: ahctf1 and kras mutations combine to amplify oncogenic stress and restrict liver overgrowth in a zebrafish model of hepatocellular carcinoma
Source: eLife. 2023 Jan 17;12:e73407. doi: 10.7554/eLife.73407 (PMC9897728; doi:10.7554/eLife.73407)
Supplement: Transparent reporting form [file elife-73407-transrepform1.pdf]

eLife's transparent reporting form

We encourage authors to provide detailed information *within their submission* to facilitate the interpretation and replication of experiments. Authors can upload supporting documentation to indicate the use of appropriate reporting guidelines for health-related research (see EQUATOR Network), life science research (see the BioSharing Information Resource), or the ARRIVE guidelines for reporting work involving animal research. Where applicable, authors should refer to any relevant reporting standards documents in this form.

If you have any questions, please consult our Journal Policies and/or contact us: editorial@elifesciences.org.

Sample-size estimation

- You should state whether an appropriate sample size was computed when the study was being designed
- You should state the statistical method of sample size computation and any required assumptions
- If no explicit power analysis was used, you should describe how you decided what sample (replicate) size (number) to use

Please outline where this information can be found within the submission (e.g., sections or figure legends), or explain why this information doesn't apply to your submission:

Sample sizes are detailed in the figure legends. In compliance with the regulations of the Walter and Eliza Hall Institute of Medical Research Animal Ethics Committee, the numbers of zebrafish larvae up to 7 dpf used in our research are not counted or recorded. Large numbers of zebrafish larvae up to 7 dpf were used in our experiments, with no ethical requirements to limit numbers. Accordingly, no explicit power analysis was used. Sample size for each measurement was determined by the practical limitations of the protocol used.

Replicates

- You should report how often each experiment was performed
- You should include a definition of biological versus technical replication
- The data obtained should be provided and sufficient information should be provided to indicate the number of independent biological and/or technical replicates
- If you encountered any outliers, you should describe how these were handled
- Criteria for exclusion/inclusion of data should be clearly stated
- High-throughput sequence data should be uploaded before submission, with a private link for reviewers provided (these are available from both GEO and ArrayExpress)

Please outline where this information can be found within the submission (e.g., sections or figure legends), or explain why this information doesn't apply to your submission:

All relevant information about replicates can be found within figure legends. We used up to three technical replicates of three to thirty biological replicates per experimental group per experiment. Each biological replicate represents an individual animal, a micro-dissected liver or a liver section from an individual animal. Each experiment was performed independently 2-3 times. All data points were included.

eLife Sciences Publications, Ltd is a limited liability non-profit non-stock corporation incorporated in the State of Delaware, USA, with company number 5030732, and is registered in the UK with company number FC030576 and branch number BR015634 at the address Westbrook Centre, Milton Road, Cambridge CB4 1YG.

Statistical reporting

- Statistical analysis methods should be described and justified
- Raw data should be presented in figures whenever informative to do so (typically when N per group is less than 10)
- For each experiment, you should identify the statistical tests used, exact values of N, definitions of center, methods of multiple test correction, and dispersion and precision measures (e.g., mean, median, SD, SEM, confidence intervals; and, for the major substantive results, a measure of effect size (e.g., Pearson's r, Cohen's d)
- Report exact p-values wherever possible alongside the summary statistics and 95% confidence intervals. These should be reported for all key questions and not only when the p-value is less than 0.05.

Please outline where this information can be found within the submission (e.g., sections or figure legends), or explain why this information doesn't apply to your submission:

Full information on statistical approaches is stated in the Materials and Methods section "Statistical Analysis". All graphs display the mean and standard error of the mean (SEM). The statistical test used, and N values are reported in the figure legends. Exact p-values are stated in figures.

Group allocation

- Indicate how samples were allocated into experimental groups (in the case of clinical studies, please specify allocation to treatment method); if randomization was used, please also state if restricted randomization was applied
- Indicate if masking was used during group allocation, data collection and/or data analysis

Please outline where this information can be found within the submission (e.g., sections or figure legends), or explain why this information doesn't apply to your submission:

Zebrafish larvae were randomly assigned to experimental and control groups. The hepatocytes analysed in each experiment were randomly sampled from the total liver section. Investigators were not blinded.

Additional data files ("source data")

- We encourage you to upload relevant additional data files, such as numerical data that are represented as a graph in a figure, or as a summary table
- Where provided, these should be in the most useful format, and they can be uploaded as "Source data" files linked to a main figure or table
- Include model definition files including the full list of parameters used
- Include code used for data analysis (e.g., R, MatLab)
- Avoid stating that data files are "available upon request"

Please indicate the figures or tables for which source data files have been provided:

Source data files have been provided for Figures 1-7.
